# Differential Trafficking Phenotypes of NPC1 Mutant Proteins Reveal Distinct Cholesterol Accumulation Profiles

**DOI:** 10.1002/jimd.70186

**Published:** 2026-04-15

**Authors:** Sanaa Abdelmalek Mahmoud, AhmedElmontaser Mergani, Maren von Köckritz‐Blickwede, Hassan Y. Naim

**Affiliations:** ^1^ Institute of Biochemistry, University of Veterinary Medicine Hannover Hannover Germany; ^2^ Research Center for Emerging Infections and Zoonoses (RIZ), University of Veterinary Medicine Hannover Hannover Germany

**Keywords:** cholesterol, filipin, lipid rafts, lysosomal storage disorder, Niemann‐Pick type C, NPC1 mutations, protein trafficking

## Abstract

Niemann‐Pick disease type C (NPC) is a rare autosomal recessive lysosomal storage disorder that affects approximately 1 in 100 000 live births. It is primarily caused by mutations in the *NPC1* gene, which disrupts intracellular cholesterol transport and leads to lipid accumulation in late endosomes and lysosomes. This results in visceral dysfunction and progressive neurological deterioration. This study investigated the effects of three NPC1 disease‐causing mutations (p.I1061T, p.D874V, and p.P1007A) on NPC1 biosynthesis, trafficking of NPC1, and cellular cholesterol levels. By expressing NPC1 mutants and wild‐type NPC1 in *NPC1* knockout Chinese hamster ovary cells (CHO‐CT43), we analysed the trafficking patterns using Western blot based on endoglycosidase H sensitivity. We also examined lipid raft distribution and unesterified cholesterol accumulation using flotillin 2, filipin staining, and high‐performance liquid chromatography (HPLC). Our findings revealed significant differences in NPC1 mutant expression levels and trafficking patterns, categorizing them into three distinct phenotypes: intracellularly retained, slow‐trafficking, and finally wild‐type‐like mutants. Notably, the variations in NPC1 mutant expression and trafficking patterns in CHO‐CT43 cells correlated with alterations in lipid rafts distribution and cellular cholesterol levels. The study demonstrated a clear association between cholesterol accumulation and NPC1 mutant trafficking phenotypes, with the p.I1061T variant exhibiting the most severe biochemical profile and highest cellular cholesterol levels. In conclusion, this study highlights a promising framework for elucidating the genotype–phenotype relationships in NPC and assessing their potential pathogenicity. These findings have significant implications for understanding the molecular mechanisms underlying NPC, and they propose personalized, targeted therapeutic strategies for this devastating disease.

## 1 Introduction

Niemann‐Pick disease type C (NPC) is a rare, autosomal recessive, lysosomal storage disorder with an incidence of approximately 1:100000 live births [1]. NPC is an extremely heterogeneous disease with clinical manifestations that may be visceral, neurological, and/or psychiatric. The disease spectrum ranges from a rapidly fatal neonatal cholestatic form to a slowly progressive adult‐onset neurodegenerative form. NPC is caused by mutations in either *NPC1* or *NPC2* genes, with approximately 95% of cases resulting from variants in the *NPC1* gene [2, 3, 4].

The *NPC1* gene encodes a large transmembrane glycoprotein of 1278 amino acids with an apparent molecular weight of 170–190 kDa with 13 transmembrane domains and 14 potential asparagine residues for N‐linked glycosylation [5, 6]. NPC1 possesses three major luminal domains: an N‐terminal domain, a middle luminal domain, and a C‐terminal cysteine‐rich domain. The N‐terminal domain has been shown to contain a cholesterol‐binding site. In addition, five of the transmembrane domains form a sterol‐sensing domain (SSD), which shows homologies to key regulators of cholesterol homeostasis [7, 8]. NPC1 protein is synthesized and N‐glycosylated in the endoplasmic reticulum (ER), and subsequently trafficked through the Golgi complex for further modifications [9]. The mature, complex‐glycosylated protein then exits the *trans*‐Golgi network and is targeted to late endosomes/lysosomes (LE/L) compartments via multiple signals including a dileucine motif within its cytoplasmic tail and an unidentified signal residing within the SSD [9, 10].

NPC1 is crucial for the regulation and intracellular trafficking of cholesterol. The low pH environment within the LE/L lumen enhances the binding of unesterified cholesterol, derived from low‐density lipoprotein, to NPC2, a small soluble glycoprotein [11, 12]. NPC2 then transfers this cholesterol to NPC1, which in turn facilitates its egress from the LE/L compartments, allowing for its redistribution to various subcellular pools [8, 13].

Cells harboring loss‐of‐function variants in the *NPC1* gene exhibit defective cholesterol trafficking and, consequently, impaired cholesterol homeostasis. This defect leads to the accumulation of a wide range of lipids within LE/L, including LDL‐derived unesterified cholesterol and sphingolipids [1, 8, 14]. The resulting accumulation disrupts the integrity of lipid rafts (LRs), specialized membrane microdomains enriched with cholesterol and sphingolipids that are vital for various cellular processes, including signal transduction, protein sorting, and membrane trafficking [15], as previously demonstrated [16].

To date, the Human Gene Mutation Database (HGMD, Qiagen) has reported over 800 disease‐causing variants in the *NPC1* gene. The molecular mechanisms underlying NPC1 disease have been proposed to involve protein folding, trafficking defects, and increased protein instability [17, 18, 19]; however, further studies are required to fully elucidate these mechanisms.

In a previous study, Shammas et al. [16] expressed a group of common *NPC1* gene variants in COS‐1 cells to analyze their pathogenicity at the biochemical level. The study categorized the NPC1 mutants into three biochemical phenotypes: normally trafficked (wild‐type like), partially trafficked, and intracellularly retained within the ER. Subsequently, Brogden et al. [20] demonstrated that lipid accumulation in NPC fibroblasts was variant‐dependent and correlated with the trafficking pattern of NPC1. However, the precise relationship between NPC1 trafficking defects and cholesterol distribution in different cellular compartments remains incompletely understood, highlighting the need for further investigation.

In the present study, we investigated the implications of three NPC1 variants on NPC1 biosynthesis, intracellular trafficking, and cellular cholesterol levels, as well as their genotype‐biochemical phenotype correlations. The NPC1 mutants were expressed in a homozygous manner in NPC1‐knockout cellular model, allowing a clearer assessment of the impact of each individual variant. Our data revealed a direct correlation between cholesterol accumulation, protein expression levels, and the trafficking phenotype of the NPC1 mutants. These findings provide a promising framework for understanding the molecular and cellular pathogenesis of NPC.

## 2 Material and Methods

### 2.1 Cell Culture and Transfection

Cell culture consumables were purchased from Sarstedt (Nümbrecht, Germany). Wild‐type Chinese hamster ovary (CHO‐WT) cells and NPC1‐knockout CHO (CT43) cells (generously provided by T. Y. Chang Dartmouth Medical School, Hanover, NH, USA) were cultured in RPMI 1640 medium (Thermo Fisher Scientific, Waltham, MA, USA) supplemented with 10% fetal calf serum (Gibco BRL, Grand Island, NY, USA) (v/v), 100 U/mL penicillin, and 100 μg/mL streptomycin (Sigma Aldrich, Darmstadt, Germany) at 37°C in a humidified incubator containing 5% CO_2_ and 95% air.

FLAG‐tagged plasmids encoding wild‐type NPC1 (NPC1‐WT) as well as NPC1 harbouring the mutations p.I1061T, p.P1007A, and p.D874V (Table 1) were transiently expressed into CT43 cells using Lipofectamine LTX with Plus Reagent (Thermo Fisher Scientific, Waltham, MA, USA). Cells were seeded in a 6 cm culture plate 1 day prior to transfection at 70%–90% confluence. The transfection was performed according to the manufacturer's instructions with slight modifications. Briefly, 3 μg of cDNA was diluted in 150 μL Opti‐MEM medium (Gibco BRL, Grand Island, NY, USA), then added to the diluted lipofectamine in 250 μL Opti‐MEM medium, incubated for 30 min and finally added to the cells. A complete RPMI 1640 media was added to the cells 3 h post‐transfection.

**TABLE 1:** Niemann‐Pick C 1 mutants analyzed in this study.

Nucleotide change	Location	Amino acid exchange	Domain
3182T>C	Exon 21	p.I1061T	Cysteine‐rich luminal loop between TM‐8 and TM‐9
3019C>G	Exon 20	p.P1007A	Cysteine‐rich luminal loop between TM‐8 and TM‐9
2621A>T	Exon 18	p.D874V	Cysteine‐rich luminal loop between TM‐8 and TM‐9

### 2.2 Cell Lysis, Immunoprecipitation and Endoglycosidase H Treatment

The cells were lysed 48 h post‐transfection for 1 h at 4°C with Triton X‐100 lysis buffer (1% Triton X‐100 in 10 mM Tris–HCl and 150 mM NaCl, pH 7.4) containing a cocktail of protease inhibitors (PI). Cellular lysates were then centrifuged at 17 000×*g* for 20 min at 4°C to remove cellular debris. Protein concentrations were calculated using Bradford reagent (Bio‐Rad Laboratories GmbH, Munich, Germany).

Further, the NPC1‐WT and NPC1 mutants were immunoprecipitated using monoclonal mouse anti‐FLAG antibody (Sigma Aldrich, Darmstadt, Germany) followed by Protein A‐Sepharose beads. The immunoprecipitated proteins were treated with endoglycosidase H (Endo H) to identify their biosynthetic structural forms. First, the immunoprecipitants were denatured using 5% SDS and 10% 2‐mercaptoethanol for 1 h at 37°C. Then, they were treated with 5 U/mL Endo H (Sigma Aldrich, Darmstadt, Germany) for 90 min at 37°C in 2.5 μL G5 buffer (0.5 M sodium citrate, pH 5.5).

The cellular lysates and the Endo H treated or untreated immunoprecipitates were mixed with Laemmli buffer containing 100 mM dithiothreitol (DTT) and analyzed by sodium dodecyl sulfate‐polyacrylamide gel electrophoresis (SDS‐PAGE), followed by Western blotting.

### 2.3 Isolation of Lipid Rafts

Lipid rafts (LRs) were isolated from CHO‐WT and CT43 cells transiently expressing NPC1 mutants 72 h post‐transfection by ultracentrifugation. The transfected cells were solubilised for 2 h at 4°C in Triton X‐100 lysis buffer supplemented with PI mixture after being homogenised by passing 20 times through a 21 G needle. The lysates were then centrifuged at 10 000×*g* for 10 min at 4°C to remove the cell debris. The supernatants were transferred into ultracentrifuge tubes and centrifuged at 100 000×*g* for 1 h at 4°C using a Type‐100 Ti rotor (Beckman Coulter, Mississauga, ON, Canada). This procedure retains the LRs in the pellet, while the non‐LRs (N‐LR) are found in the supernatant [21]. The pellet was resuspended in 25 mM Tris‐buffer (pH 8) containing 50 mM NaCl, 0.5% sodium deoxycholate and 0.5% Triton X‐100 (same volume as the supernatant). Similar volumes of LRs and N‐LRs were then subjected to SDS‐PAGE, followed by Western blotting to examine the distribution of the LR protein marker, flotillin‐2 (FLOT2).

### 2.4 SDS‐PAGE and Western Blot

Protein samples were denatured at 37°C for 1 h and analyzed by SDS‐PAGE under reducing conditions on 8% gels. The resolved proteins were transferred onto a polyvinylidene difluoride (PVDF) membrane (Merck KGAA, Darmstadt, Germany), and the membrane was blocked overnight in 5% skimmed milk in 0.1% Tween‐PBS at 4°C. The PVDF membrane was then incubated with appropriate primary antibody diluted in 2% skimmed milk in 0.1% Tween‐PBS for 1 h. After washing the membrane 3 times with 0.1% Tween‐PBS, the secondary antibody conjugated to horseradish peroxidase was added for 45 min, followed by another 3 washes. All antibodies used are listed in Table 2. Protein bands were detected via enhanced chemiluminescence (ECL) with the SuperSignal West Femto Maximum Sensitivity Substrate (Thermo Fisher Scientific, Waltham, MA, USA), visualised by the ChemiDoc MP Touch Imaging System, and quantified using Image Lab Software 5.2.1 (Bio‐Rad Laboratories GmbH, Munich, Germany).

**TABLE 2:** Primary and secondary antibodies.

Antibody	Concentration	Dilution	Company/Cat #
Rabbit anti‐NPC1	1 μg/μL	1:3000	Novus Biologicals, NB400‐148
Mouse anti‐FLAG M2	1 μg/μL	1:5000	Sigma‐Aldrich, F1804‐1MG
Beta‐Actin	0.2 μg/μL	1:5000	Santa Cruz Biotechnology, sc‐47 778
Flotillin‐2	0.2 μg/μL	1:5000	Santa Cruz Biotechnology, sc‐28 320
Goat anti‐mouse IgG secondary antibody HRP	0.4 μg/μL	1:5000	ThermoFisher Scientific, 31 430
Goat anti‐rabbit IgG secondary antibody HRP	0.4 μg/μL	1:5000	ThermoFisher Scientific, 31 460

### 2.5 Filipin Staining and Fluorescence Microscopy

The FLAG‐tagged NPC1 mutants were transiently transfected into CT43 cells, which were seeded in coverslips 24 h prior to transfection. All the following steps were performed at room temperature. Forty‐eight hours post‐transfection, the cells were washed twice with PBS, fixed with 4% paraformaldehyde for 30 min, and washed thereafter twice with PBS. Fixed cells were quenched with 50 mM ammonium chloride for 30 min and then stained for 2 h in the dark with Filipin (5 mg/mL, Sigma Aldrich, Darmstadt, Germany) diluted 1:50 in PBS. After washing twice with PBS, cells were mounted with Mowiol in combination with the anti‐bleaching reagent DABCO (Roth, Karlsruhe, Germany) and visualised by a Leica DM IRB fluorescent microscope.

### 2.6 Cholesterol Analysis

NPC1 mutants were transiently expressed in CT43 cells as mentioned in section 2.1. The cells were washed twice with PBS 48 h post‐transfection, collected and resuspended in 1 mL PBS. The cells were then counted and adjusted to 1 000 000 cells, followed by homogenisation via passage through 18 G needle. Lipids were thereafter isolated using chloroform/methanol method of Bligh and Dyer [22], following the protocol previously described by Brogden et al. [20]. The samples were redissolved in 125 μL of a 1:1 chloroform/methanol solution and total cholesterol levels were measured by a Hitachi Chromaster high‐performance liquid chromatography (HPLC) system.

### 2.7 Statistical Analysis

All experiments were repeated at least three independent times. Statistical analysis was performed using GraphPad Prism Software 10.4.1 (627; GraphPad Company—Dotmatics, Boston, MA, USA). A single comparison between CHO‐WT and CT43 cells data was analyzed by a two‐tailed *t*‐test. Multiple comparisons were analyzed by one‐way ANOVA with Tukey's multiple comparisons test. Data are shown as mean ± standard error. *p* values of ≤ 0.05 were considered statistically significant. Means and standard errors were calculated by Microsoft Excel for Microsoft 365 MSO, 2502 (Microsoft, Redmond, WA, USA) software.

### 2.8 Use of Artificial Intelligence

Language corrections in the manuscript have been made by ChatGPT.

## 3 Results

### 3.1 Expression of NPC1 Mutants in CT43 Cells

**FIGURE 1:**
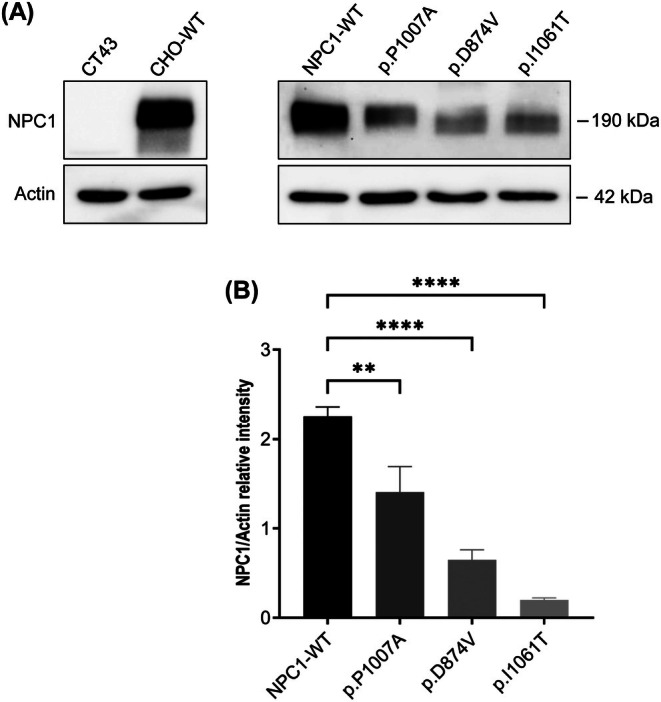
Expression of the NPC1 mutants in CT43 cells. (A) The FLAG‐tagged NPC1 mutants were transiently transfected into CT43 cells using Lipofectamine LTX with Plus Reagent and the cellular lysates were analyzed by Western blotting using anti‐FLAG antibody. (B) NPC1 was quantified and normalized to Actin. NPC1 mutants exhibited a significant reduction in protein expression in comparison to wild‐type (WT) controls. Statistical analysis was performed one‐way ANOVA. Data are shown as mean ± SEM, *n* = 6. **p ≤ 0.01, ****p ≤ 0.0001.

NPC1 mutants were transiently transfected into NPC1‐knockout CHO (CT43) cells to investigate their expression level in comparison to the wild‐type NPC1 (NPC1‐WT) protein. The cells expressing NPC1 mutants were lysed 48 h post‐transfection and analyzed by western blotting (Figure 1A,B).

The expression of the mutants in knocked‐out system allows for a better assessment of the mutated gene function by observing the effect of a single variant. NPC1‐WT appears as a single diffuse band at a molecular weight of 190 kDa. We observed a significant reduction in protein expression for the p.I1061T mutant (8.9% ± 0.1% of NPC1‐WT levels), whereas the protein level of the p.P1007A mutant was near to the NPC1‐WT (62.3% ± 0.7%). The p.D874V mutant exhibited an intermediate level of protein expression between the p.I1061T and p.P1007A mutants (28.8% ± 0.3% of NPC1‐WT levels). These findings suggest that the p.I1061T mutation significantly impairs protein stability or folding, leading to reduced protein expression. In contrast, the p.P1007A mutation appears to have minimal effects on protein expression, suggesting that this mutation may not significantly impact NPC1 function.

### 3.2 Distinct Biosynthetic Forms of NPC1 Mutants Define Their Intracellular Trafficking Patterns

**FIGURE 2:**
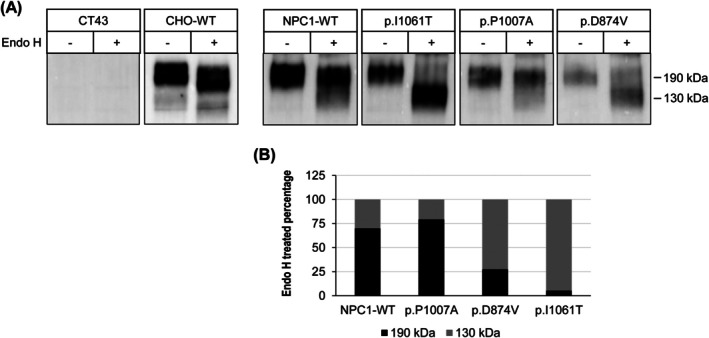
Biosynthetic forms of the NPC1 mutants and their trafficking pattern. The FLAG‐tagged NPC1 mutants were transiently expressed in CT43 cells. The NPC1 mutants were immunoprecipitated with anti‐FLAG antibodies, treated (+) or non‐treated (−) with Endo H and analyzed by Western blotting to assess their biosynthetic forms. The Endo H‐resistant mature form appears as a 190 kDa band and the mannose‐rich Endo H‐sensitive immature form is cleaved to a 130 kDa band. NPC1 mutants were categorized into three forms: Non‐trafficked/ER‐Block (p.I1061T), partially trafficked (p.D874V), and normally trafficked/WT‐like (p.P1007A).

To elucidate the biosynthetic structural forms and trafficking patterns of the NPC1 mutants, we treated the immunoprecipitates with endoglycosidase H (Endo H). This enzyme specifically cleaves immature N‐linked high‐mannose glycans of glycoproteins in the ER, allowing us to distinguish between different biosynthetic forms [19]. Our analysis reveals that wild‐type NPC1 protein exhibits two characteristic biosynthetic forms, a mannose‐rich, Endo H‐sensitive form (NPC1h) localized in the endoplasmic reticulum (ER) and a complex glycosylated, Endo H‐resistant form (NPC1c) processed in the Golgi and trafficked to lysosomes. These two forms, which appeared together as a broad diffuse band, could only be resolved from each other after Endo H treatment. With this approach we were able to categorize the NPC1 mutants into three distinct groups based on their trafficking patterns: completely non‐trafficked (ER‐Block), partially trafficked and normally trafficked (wild type like) as previously described by [16]. Notably, our data show that the NPC1 protein harboring the p.P1007A mutation exhibits a biosynthetic pattern nearly indistinguishable from that of the wild‐type NPC1 protein, suggesting that this mutation does not significantly impair protein trafficking through the Golgi (Figure 2A,B). In sharp contrast, the p.I1061T mutant is completely blocked in the ER, as confirmed by its sensitivity to Endo H treatment and pronounced shift to a lower apparent molecular weight (130 kDa). This finding indicates that the p.I1061T mutation severely disrupts protein trafficking, leading to ER retention. Finally, the NPC1 containing the p.D874V mutation displays partial resistance to Endo H treatment concomitant with a delayed exit of this mutant from the ER. This suggests that the p.D874V mutation impairs, but does not completely block, protein trafficking, resulting in a mixed biosynthetic profile.

### 3.3 Variable Levels of Lipid Rafts Triggered by NPC1 Mutations are Associated With Their Biosynthetic Forms

**FIGURE 3:**
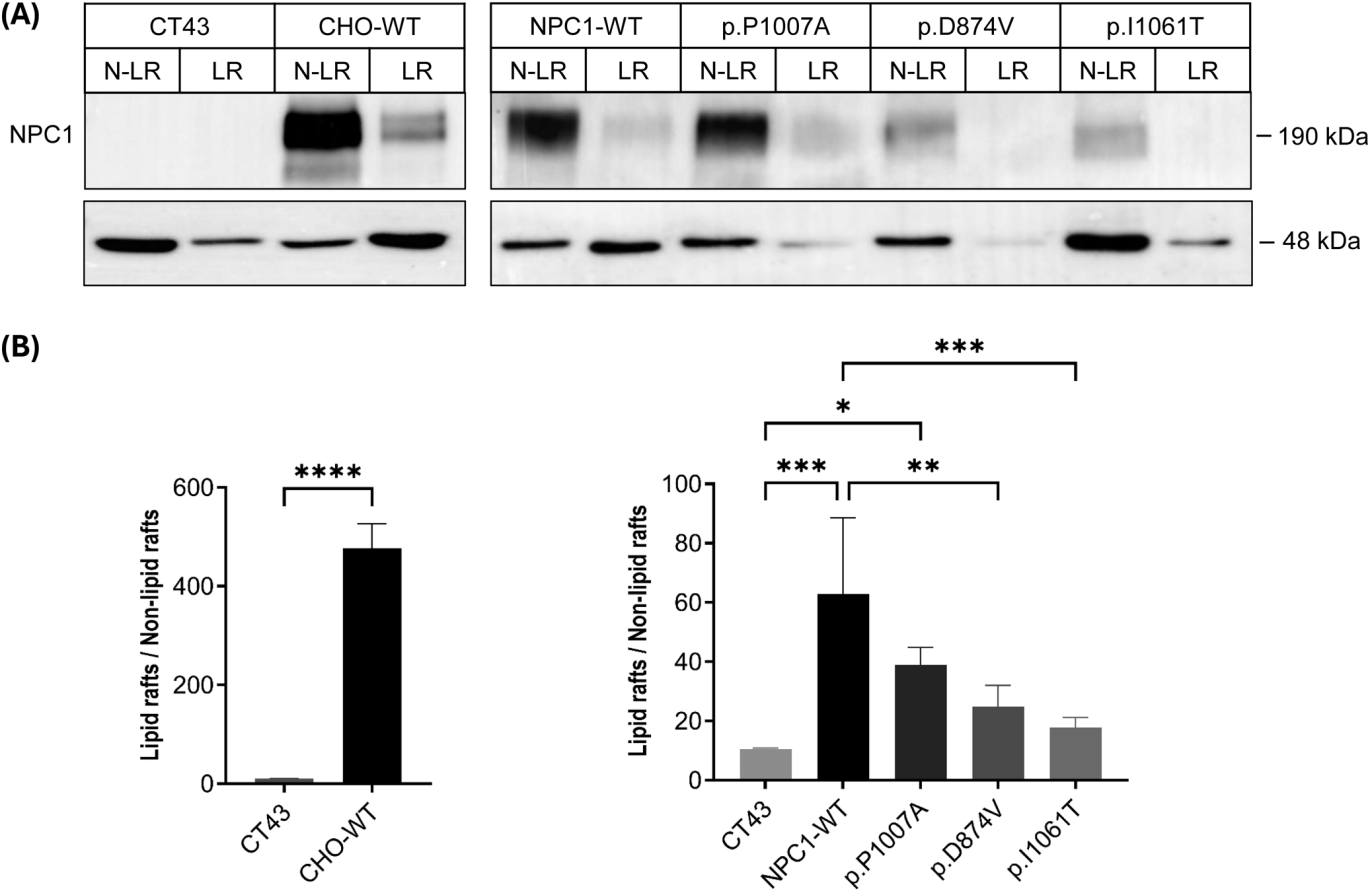
Lipid raft associated proteins in CT43 cells expressing NPC1 mutants. (A) Lipid rafts were isolated from CT43 cells transiently expressing NPC1 mutants 72 h post‐transfection by ultracentrifugation and analyzed by Western blotting. (B) Flotillin‐2, a lipid rafts marker, was quantified and the lipid rafts (LR) to non‐lipid rafts (N‐LR) ratio was statistically analyzed by *t*‐test or one‐way ANOVA. A significant decrease was observed in the LRs in the cells expressing NPC1 mutants harboring the p.I1061T and p.D874V variants, while the NPC1 mutant bearing the p.P1007A variant showed a nominal but non‐significant decrease as compared to wild‐type (WT) controls. Data are shown as mean ± SEM, *n* = 4. *p ≤ 0.05, **p ≤ 0.01, ***p ≤ 0.001, ****p ≤ 0.0001.

To investigate the effects of the differential trafficking of the NPC1 mutants on the cellular cholesterol levels, we examined the distribution of flotillin‐2 in cholesterol‐ and sphingolipids‐enriched membrane microdomains, known as lipid rafts (LRs). LRs were isolated from CT43 cells transiently expressing NPC1 mutants 72 h post‐transfection by ultracentrifugation, which resulted in the LRs being concentrated in the pellet. Western blot analysis revealed a significant reduction in the distribution of flotillin‐2 in the pellets derived from the cells expressing NPC1 mutants as compared to the wild‐type controls (Figure 3A,B). Notably, the pattern of reduction of flotillin‐2 distribution correlated with the biosynthetic forms of the NPC1 mutants. The I1061T substitution, which was found to be retained in the ER and exhibited the most severe biochemical profile, resulted in a highly significant decrease in flotillin‐2 distribution in the LRs, suggesting a profound disruption of LRs formation. In contrast, cells expressing the p.P1007A mutation, which displayed a similar trafficking pattern to NPC1‐WT, showed only a slight reduction in flotillin‐2 distribution. The partially trafficked p.D874V mutant, however, triggered a moderate reduction in flotillin‐2 distribution in LRs, intermediate between the comparison to the p.I1061T and p.P1007A mutants. These findings suggest that the biosynthetic forms of NPC1 mutants influence the formation and stability of lipid rafts.

### 3.4 Differential Cellular Cholesterol Accumulation Correlates With Variable Expression and Trafficking Patterns of the NPC1 Mutants

**FIGURE 4:**
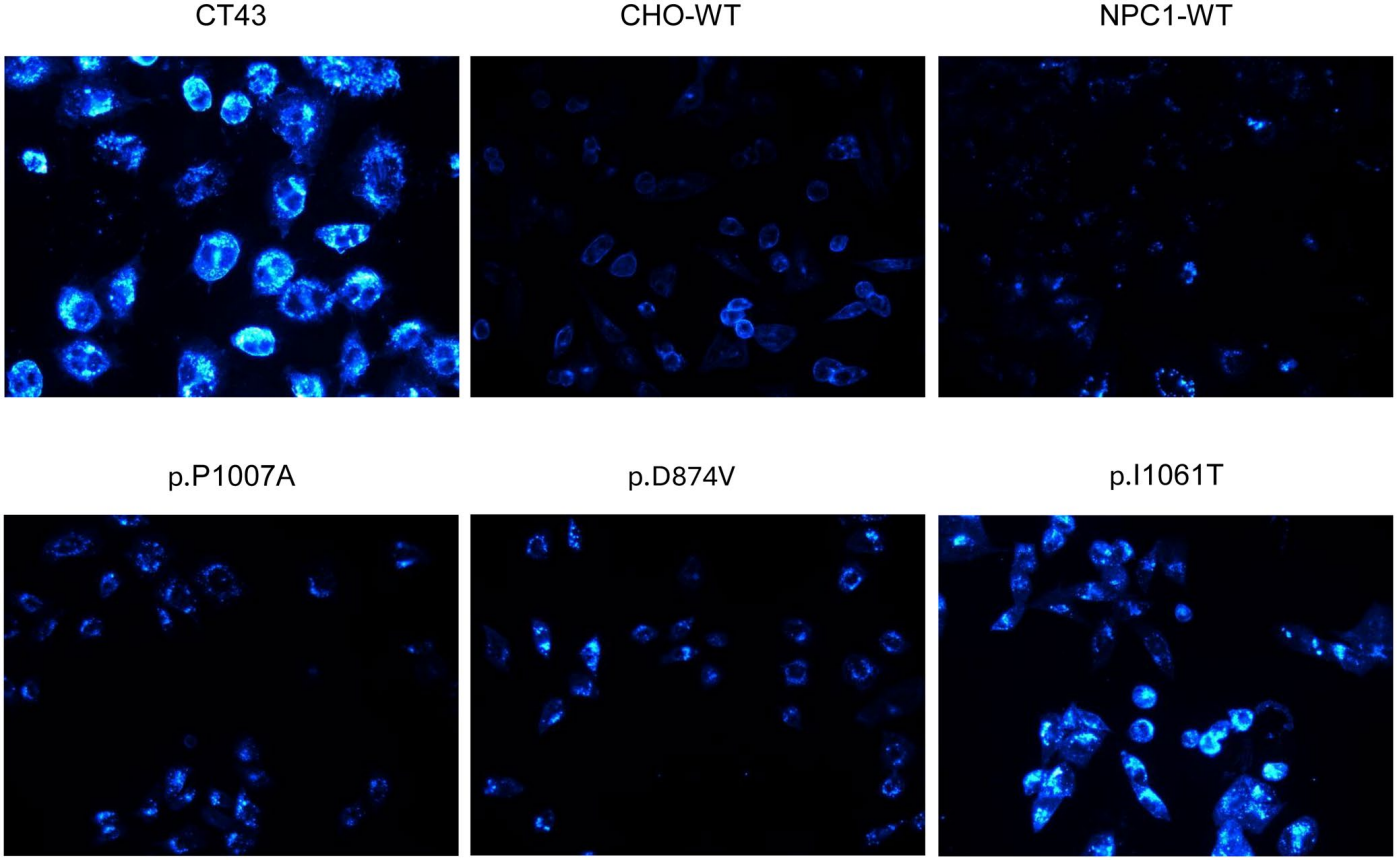
Assessment of cholesterol accumulation by filipin staining. The NPC1 mutants were transiently expressed in CT43 cells using Lipofectamine LTX with Plus Reagent. The cells were stained 48 h post‐transfection with filipin and visualized by fluorescence microscope to visualize the cholesterol storage. Fluorescence microscopic images demonstrate variations in cholesterol accumulation in the CT43 cell expressing NPC1 mutants compared to controls. Scale bar: 50 μm.

**FIGURE 5:**
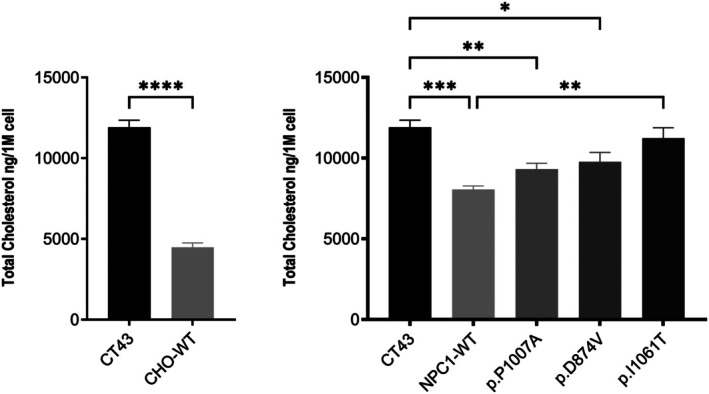
Measurement of cellular cholesterol in CT43 cells expressing NPC1 mutants. The NPC1 mutants were transiently transfected into CT43 cells. Lipids were isolated using a chloroform/methanol‐based method and total cholesterol levels were measured by high‐performance liquid chromatography (HPLC). NPC1 mutants triggered an increase in cellular cholesterol levels as compared to wild‐type (WT) controls, but this increase was only significant for the NPC1 mutants carrying the p.I1061T variant. Statistical analysis was carried out by *t*‐test or one‐way ANOVA. Data are shown as mean ± SEM, *n* = 4–6. *p ≤ 0.05, **p ≤ 0.01, ***p ≤ 0.001, ****p ≤ 0.0001.

**FIGURE 6:**
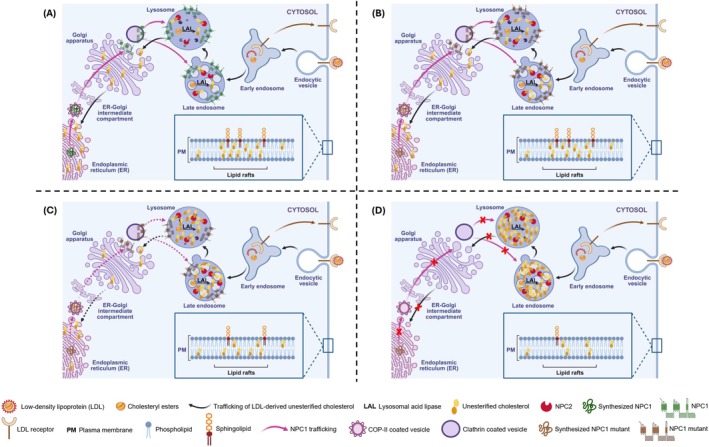
A schematic presentation that reveals a direct correlation between the NPC1 biochemical phenotype, cellular cholesterol accumulation, and lipid raft variations. The NPC1 protein is synthesized and N‐glycosylated in the endoplasmic reticulum, then trafficked to the Golgi complex via COPII vesicles, where it undergoes complex glycosylation and maturation before being transported to lysosomes. NPC1 and luminal NPC2 collaborate to export cholesterol from the lysosomes into the cytoplasm, maintaining cholesterol homeostasis as well as cholesterol‐ and sphingolipid‐enriched membrane microdomains, or lipid rafts (as shown in the inset, zoomed out of the cell surface; for simplicity, only sphingolipids and cholesterol are depicted, excluding associated membrane proteins). (A) Trafficking of wild‐type NPC1, with intracellular normal cholesterol levels and intact lipid rafts. (B) The p.P1007A variant in NPC1 leads to a slight elevation of cholesterol levels and alteration in lipid rafts, despite a normal trafficking pattern. (C) NPC1 mutant carrying the p.D874V variant reveals an overall delayed trafficking pattern and an intermediate increase in cholesterol levels, accompanied by lipid raft alteration. (D) NPC1 mutant harboring the p.I1061T variant is retained in the endoplasmic reticulum and demonstrates the highest cholesterol accumulation and most pronounced lipid raft distortion. This figure was created using BioRender.com.

To investigate the impact of NPC1 mutations on cholesterol accumulation, we employed the fluorescent dye Filipin to visualize cholesterol distribution in CT43 expressing NPC1 mutants. Filipin staining revealed distinct variations in cholesterol accumulation among the CT43 cells expressing NPC1 mutants as compared to the wild‐type controls (Figure 4). To quantify cellular cholesterol levels, we isolated lipids 48 h post‐transfection using a chloroform/methanol‐based method and total cellular cholesterol levels were then measured by high‐performance liquid chromatography (HPLC). Consistent with Filipin staining results, HPLC analysis revealed a significant increase in cellular cholesterol levels in cells expressing the NPC1 mutants compared to those expressing wild‐type NPC1 (Figure 5). Notably, the extent of cholesterol accumulation correlated with the level of NPC1 expression and trafficking pattern (Figure 6). In fact, the NPC1‐knockout cells exhibited a 2.5‐fold higher cholesterol level compared to CHO‐WT cells, similar to that observed in cells expressing the non‐trafficked p.I1061T mutant. This finding highlights the critical role of NPC1 in maintaining cholesterol homeostasis and suggests that the p.I1061T mutant is functionally equivalent to an NPC1‐null state. In contrast, cells expressing both normally and partially trafficked mutants, p.P1007A and p.D874V respectively, displayed lower levels of cholesterol accumulation, indicating that these mutants retained some residual function in mediating some cholesterol efflux. Altogether, these results demonstrate that the level of NPC1 expression and trafficking pattern are key determinants of cholesterol accumulation in cells expressing NPC1 mutants.

## 4 Discussion

The mutational spectrum is extremely heterogeneous in the NPC1 disease. Over 800 disease‐causing mutations have been identified in the lysosomal transmembrane glycoprotein, NPC1. These mutations showed a wide distribution among NPC1 domains, with a majority existing in the conserved NPC1 cysteine‐rich luminal domain [23]. Several mutational studies have been conducted in NPC fibroblasts during the last three decades aiming to characterize the molecular basis of NPC1 disease and establish a genotype–phenotype correlation. However, few studies have investigated the significance of single variants within the *NPC1* gene in NPC1 knocked‐out models.

In this article, we selected three NPC1 missense mutants that are commonly predicted to cause NPC1 disease based on the diversity of the trafficking phenotypes of the NPC1 mutants described by Shammas et al. [16]. Our goal was to define their impact on the NPC1 biosynthesis, trafficking and functionality after transfection into *NPC1* knocked‐out CHO cells (CT43). Our findings revealed substantial differences in the expression levels and trafficking patterns of NPC1 mutants, as well as cellular cholesterol levels and lipid rafts. Notably, the p.I1061T is the most prevalent NPC1 mutant and has been reported in approximately 20% of patients of European descent [24, 25]. We found that NPC1 protein harboring the mutation p.I1061T was completely blocked in the endoplasmic reticulum (ER) and exhibited a highly significant reduction in protein expression level. Furthermore, this mutant resulted in a highly significant elevation in the cellular cholesterol levels and decrease in the lipid rafts marker protein, flotillin‐2. These results are consistent with the previous observations that the p.I1061T substitution was associated with severe impairment of cholesterol trafficking.

The level of NPC1 protein in fibroblasts from homozygous I1061T patients varied from partially to very slightly diminished [23, 25, 26]. Another study by Gelsthorpe et al. [17] showed that NPC1 protein levels are decreased by 85% in human fibroblasts homozygous for the p.I1061T mutation and it remains Endo H‐sensitive, indicating an endoplasmic reticulum trafficking defect as also confirmed by Schultz et al. [19]. These data suggested that p.I1061T mutant protein is targeted to the ER quality control due to protein misfolding [17, 19]. In contrast to the p.I1061T substitution, our assessment of the CT43 cells expressing the p.P1007A variant showed similarity in protein expression level and trafficking pattern to that of the NPC1‐WT. As expected, this mutant exhibited only a slight increase in cellular cholesterol accumulation and a slight reduction in flotillin‐2.

The p.P1007A mutant is initially described in homozygosity in a Canadian patient and has been associated with a mild NPC biochemical phenotype [24], which is consistent with our results. Additionally, previous studies revealed minimal accumulation of unesterified cholesterol and no significant alterations of the cholesterol homeostasis in NPC1 patients carrying the p.P1007A mutant [14, 23, 26]. Ribeiro et al. [26] also observed a normal level of mature NPC1 protein in fibroblasts from adult‐onset patients harboring this mutant. Furthermore, we found that the NPC1 containing the p.D874V mutant was partially trafficked through the Golgi and exhibited an intermediate level of protein expression and cellular cholesterol, positioned between the p.I1061T and p.P1007A mutants. Similarly, the reduction in the flotillin‐2 triggered by this mutant was moderate.

The p.D874V mutant has been reported in the homozygous state and led to the classic biochemical phenotype [23]; however, the exact severity of this variant was not mentioned. Overall, our results in protein trafficking confirm the findings of Shammas et al. [16] that the NPC1 mutants can be classified into three major trafficking phenotypes: normally trafficked‐wild type‐like (p.P1007A), partially trafficked (p.D874V) and intracellularly blocked in the endoplasmic reticulum (p.I1061T). Interestingly, the lipid and protein analyses in this study reveal a direct correlation between trafficking of NPC1 protein and cholesterol accumulation in the cell model used. It is evident that the p.I1061T substitution resulted in the most severe biochemical profile concomitant with the highest levels of cellular cholesterol and lowest levels of lipid rafts. On the contrary, the cellular cholesterol levels were moderately increased and almost similar to wild‐type NPC1 in the cells expressing p.D874V and p.P1007A variants, respectively. This agrees with the observation of Brogden et al. [20] that lipid accumulation in fibroblasts derived from NPC1 patients was variant‐dependent and correlated with the trafficking pattern of NPC1 protein. Moreover, an earlier mutational study by Ribeiro et al. [26] indicated that the mutational profile appeared to be correlated with the biochemical phenotype, that is, severe and mild cholesterol traffic impairment. The information regarding correlations between genotype and biochemical phenotypes in NPC1 patients remains limited, mainly due to the private nature of most NPC1 mutations, as stated by Dardis et al. [27].

This study provides a comprehensive understanding of the interrelationship between protein phenotype, cholesterol content, and pathogenesis in Niemann‐Pick disease type C1 (NPC1). By examining three NPC1 missense mutants in *NPC1* knocked‐out CHO cells, we demonstrated that the trafficking patterns and expression levels of these mutants significantly impact cellular cholesterol levels and lipid rafts. These findings suggest that alterations in lipid rafts, which play crucial roles in a variety of cellular processes [15] and are closely tied to cholesterol levels, may serve as a valuable biomarker for monitoring the progression and severity of NPC.

The study highlights the importance of genotype–phenotype correlations in NPC1 disease. The findings reveal a direct correlation between NPC1 protein trafficking and cholesterol accumulation, with the p.I1061T mutant exhibiting the most severe biochemical profile and the p.P1007A mutant showing a mild phenotype. Notably, the biochemical phenotype of the p.P1007A variant is in line with a mild clinical presentation. However, the biochemical severity of the I1061T mutant protein appears inconsistent with the relatively mild clinical phenotype observed in homozygous patients, suggesting that further investigation is needed to elucidate the underlying mechanisms. In conclusion, our study has significant implications for understanding the pathogenesis of NPC1 disease and the development of therapeutic strategies. By identifying correlations between genotype and biochemical phenotypes, the severity of the disease can be better predicted with the ultimate goals of developing personalized or targeted treatments. This study contributes to our understanding of the molecular mechanisms underlying NPC1 disease and highlights the importance of continued research into this devastating disorder.

## Author Contributions

S.A.M. performed experiments and statistical analysis, interpreted the results and wrote the original draft of the manuscript. A.M. worked on the HPLC analysis, reviewed and edited the manuscript. M.v.K.‐B. co‐supervised, reviewed and edited the manuscript. H.Y.N. developed the concept of the study, interpreted the results, supervised and generated the final version of the manuscript. All authors have read and approved the contents of the manuscript.

## Funding

This work was supported by intramural funds from the University of Veterinary Medicine Hannover, Hannover, Germany. Sanaa Mahmoud has been supported by a scholarship from the German Academic Exchange Service (DAAD), Bonn, Germany. The authors confirm independence from the sponsors; the content of the article has not been influenced by the sponsors.

## Ethics Statement

The authors have nothing to report.

## Conflicts of Interest

The authors declare no conflicts of interest.

## Data Availability

The data that support the findings of this study are available from the corresponding author upon reasonable request.
